# Evaluating the Association Between Single Item Literacy Screener and Health Outcomes in Patients With Lung Cancer

**DOI:** 10.1093/geroni/igaa057.1387

**Published:** 2020-12-16

**Authors:** Julie Nguyen, Caitlyn McNaughton, Jessica Sautter

**Affiliations:** 1 University of the Sciences, Philadelphia, Pennsylvania, United States; 2 Lancaster General Health, Lancaster, Pennsylvania, United States

## Abstract

Health literacy is becoming increasingly important in areas such as cancer care, where treatments are relatively difficult to navigate. This study aims to describe the how health literacy is associated with healthcare outcomes and health system usage among patients with lung cancer. Data include retrospective medical record data from 456 patients with lung cancer; half were age 70 and older. Patients were coded as having adequate or limited health literacy based on their response to their Single Item Literacy Screener (SILS). Data were collected from a 12 month period following diagnosis for each patient. One-third of patients had limited health literacy; this was significantly more common among adults age 70 and older. Patients with limited health literacy were more likely to have newly diagnosed lung cancers of stage 3B or higher (59.18% vs. 42.76%, p = 0.0011) compared to those with adequate health literacy. Patients with limited health literacy had higher median levels of depression based on the PHQ-9 questionnaire (4.0 vs. 3.0, p = 0.0082) and a higher median number of emergency department visits (1 vs. 0, p = 0.0156) and unplanned hospitalizations (1 vs. 0, p = 0.0044). Furthermore, patients with limited health literacy were more likely to have an emergency department visit or unplanned hospitalization sooner (p < 0.0001). These data illustrate a lower quality of life and a higher dependency on healthcare services for patients with limited health literacy. Assessment and interventions may be necessary to ensure access to quality healthcare.

